# Evidence of compounded disturbance effects on vegetation recovery following high-severity wildfire and spruce beetle outbreak

**DOI:** 10.1371/journal.pone.0181778

**Published:** 2017-08-04

**Authors:** Amanda R. Carlson, Jason S. Sibold, Timothy J. Assal, Jose F. Negrón

**Affiliations:** 1 Graduate Degree Program in Ecology, Colorado State University, Fort Collins, Colorado, United States of America; 2 Department of Anthropology, Colorado State University, Fort Collins, Colorado, United States of America; 3 U.S. Geological Survey, Fort Collins Science Center, Fort Collins, Colorado, United States of America; 4 U.S. Forest Service, Rocky Mountain Research Station, Fort Collins, Colorado, United States of America; Pacific Northwest National Laboratory, UNITED STATES

## Abstract

Spruce beetle (*Dendroctonus rufipennis*) outbreaks are rapidly spreading throughout subalpine forests of the Rocky Mountains, raising concerns that altered fuel structures may increase the ecological severity of wildfires. Although many recent studies have found no conclusive link between beetle outbreaks and increased fire size or canopy mortality, few studies have addressed whether these combined disturbances produce compounded effects on short-term vegetation recovery. We tested for an effect of spruce beetle outbreak severity on vegetation recovery in the West Fork Complex fire in southwestern Colorado, USA, where much of the burn area had been affected by severe spruce beetle outbreaks in the decade prior to the fire. Vegetation recovery was assessed using the Landsat-derived Normalized Difference Vegetation Index (NDVI) two years after the fire, which occurred in 2013. Beetle outbreak severity, defined as the basal area of beetle-killed trees within Landsat pixels, was estimated using vegetation index differences (dVIs) derived from pre-outbreak and post-outbreak Landsat images. Of the seven dVIs tested, the change in Normalized Difference Moisture Index (dNDMI) was most strongly correlated with field measurements of beetle-killed basal area (R^2^ = 0.66). dNDMI was included as an explanatory variable in sequential autoregressive (SAR) models of NDVI_2015_. Models also included pre-disturbance NDVI, topography, and weather conditions at the time of burning as covariates. SAR results showed a significant correlation between NDVI_2015_ and dNDMI, with more severe spruce beetle outbreaks corresponding to reduced post-fire vegetation cover. The correlation was stronger for models which were limited to locations in the red stage of outbreak (outbreak ≤ 5 years old at the time of fire) than for models of gray-stage locations (outbreak > 5 years old at the time of fire). These results indicate that vegetation recovery processes may be negatively impacted by severe spruce beetle outbreaks occurring within a decade of stand-replacing wildfire.

## Introduction

Climate-related disturbances in North American forests have been increasing in frequency and extent in recent decades [1]. In the Rocky Mountain region, the increasing pressures of novel climate conditions, prolonged droughts, insect outbreaks, and larger and more severe wildfires have sparked concerns that multiple disturbances may drive fundamental shifts in species compositions and ecosystem processes [2–4]. Events which alter recovery processes and drive ecosystems toward new stable states are known as ‘compound disturbances’ [5], and may play an important role in shaping the structure and composition of future forests [6,7]. However, evidence supporting clear compounded effects of multiple disturbances in western forest systems is not well documented. An improved understanding of interactions between disturbances is important for building an understanding of multiple disturbance processes, and for informing management decisions in systems undergoing changes in disturbance regimes.

One of the most pressing research questions related to multiple disturbances in western forest systems is whether severe bark beetle outbreaks can increase the ecological severity of subsequent wildfires [8–10]. Millions of hectares of western conifer forests have been recently affected by several species of bark beetle, including the mountain pine beetle (*Dendroctonus ponderosae*), spruce beetle (*Dendroctonus rufipennis*), western balsam bark beetle (*Dryocoetes confusus*), Douglas-fir beetle (*Dendroctonus pseudotsugae*), and pinyon Ips beetle (*Ips confusus*) [11]. These insects have caused forest mortality at an unprecedented scale over the last two decades, due in large part to warming temperatures and aging forest stands [12,13]. The spruce beetle in particular is spreading rapidly through high-elevation subalpine forests as a possible result of increasing summer temperatures, which may shorten beetle development cycles, and increasing winter temperatures, which may allow larger populations to survive [14,15]. Spruce beetles are likely to continue spreading to higher elevations and more northerly latitudes throughout the Rocky Mountain region [16].

In the southern Rockies (southern Wyoming to northern New Mexico), the primary spruce beetle host species is Engelmann spruce (*Picea engelmannii)*. *P*. *engelmannii* typically co-occurs with subalpine fir (*Abies lasiocarpa*) at elevations ranging from about 2,850–3,500 m a.s.l. [17]. Spruce-fir forests are characterized by infrequent, high-severity wildfire and fire occurrence is climate-limited rather than fuel-limited [18–22]. As a result of the typically long intervals between fires in these systems, fuels tend to be densely stocked [21,22]. However, surface fuels are often limited [23], and beetle outbreaks may affect the fuel structure of recently killed stands by transferring fine fuels from the canopy to the forest floor.

Severe spruce beetle outbreaks can cause up to 100% mortality in mature spruce stands and result in complete loss of overstory canopy [24]. During an outbreak, host trees are killed within 1–2 years of attack as beetles bore into the bark and feed on phloem tissues. Loss of canopy needles continues for 2–5 years after the initial attack, after the tree has been killed (known as the “red stage”) [24]. The red stage is followed by a “gray stage” in which all needles have been shed and fine fuels begin to decompose on the forest floor [10]. During this time, coarse surface fuel loads may increase as standing dead trees begin to fall from root rotting and blowdowns [25]. Accumulation of fuels on the forest floor can potentially increase the severity of surface fire [26], leading managers to speculate that fuel removal may be necessary to mitigate wildfire impacts [27].

Assessing the impact of bark beetle outbreaks on fire severity is challenging, due to the difficulty in accurately quantifying outbreak severity (referring to the number or density of killed trees within a stand) after fires have damaged physical evidence of beetle activity [28]. Although aerial imagery and aerial detection survey (ADS) data can be used to classify where outbreaks have occurred at broad scales, it is difficult to determine how severity may vary at fine spatial scales. ADS is carried out annually by multiple resource agencies in the US and provides classifications of severity within hand-drawn outbreak extent polygons, but these classifications provide only a single severity estimate within areas which may vary widely in size (e.g. >1,000 ha). Remotely sensed vegetation indices (VIs) derived from satellite imagery offer the potential to estimate outbreak severity with greater spatial accuracy than ADS (30-m resolution from Landsat imagery), and can be used to characterize canopy change from outbreaks over a greater spatial extent than is feasible using field methods. Remote sensing techniques have been widely applied to detect canopy change from bark beetle outbreaks and other types of disturbance [28–36]. Multi-date image differencing of VIs provides a quantitative indicator of spectral change from forest canopy mortality [37,38], which may serve as an effective metric for canopy loss from beetle outbreak.

An additional challenge in assessing the relationship between outbreak severity and fire impacts is that a number of contingent factors may affect the nature of the disturbance interaction. These factors may complicate the effect of beetle outbreaks on fuel structures, alter the effect of fuel structure on fire behavior and burning intensity, or may affect vegetation recovery independently from fuel structure. First, the fuel structure of beetle-killed stands changes with time following the initial outbreak. Older beetle-killed stands contain greater amounts of downed woody material and ladder fuels from sapling regeneration, which allow faster surface spread and increase the probability of fire spreading to the crown [39]. However, more recently killed stands may retain more fallen needles on the forest floor which increase fine surface fuel loads [40]. The effect of fuel structure on fire severity can also vary with weather conditions at the time of burning, such that extreme temperatures, humidity levels, and wind speed are more likely to result in faster fire spread and complete combustion of fuels [39,41,42]. Additionally, topographic factors influence fire behavior (e.g., fire intensity may be greater on steeper slopes or at high slope positions) [43] and spatial patterns in vegetation recovery (e.g., faster recovery on north-facing slopes due to greater moisture availability, or at lower elevations due to warmer temperatures and longer growing season) [44,45].

Several recent studies have assessed impacts of beetle outbreaks on fire severity, and many have found no evidence of a conclusive link between disturbances [39–43,46–52]. However, previous methods of assessing fire severity may not thoroughly address all potential effects on ecosystem recovery. “Fire severity” is a somewhat ambiguous term in the literature [53], and most beetle-wildfire interaction studies have primarily focused on impacts to canopy vegetation and aboveground cover immediately after the fire. These methods do not directly account for impacts to belowground soil properties which may have a longer-term effect on vegetation recovery, such as destruction of the seed bank, alteration of soil structure, loss of organic matter, or increases in hydrophobicity [54,55]. Because the primary effect of spruce beetle outbreaks on forest stands is to shift fuels from the canopy to the forest floor, it is possible that outbreaks may impact these properties without creating any significant effect on canopy mortality. Moreover, in forests characterized as having stand-replacing fire regimes where nearly all canopy trees are killed [21], it is not clear how bark beetles could exacerbate mortality associated with wildfire.

To determine whether spruce beetle outbreak severity shows an effect on short-term vegetation recovery from fire, we used the Landsat-derived Normalized Difference Vegetation Index (NDVI) to assess understory vegetation recovery two years after a large, high-severity wildfire. NDVI provides an indicator of grass and herbaceous cover in early recovery stages [44,45,56]. We chose the West Fork Complex fire in southwestern Colorado, USA, as a case study because this event exemplifies an extreme wildfire event co-occurring with severe spruce beetle disturbance. The goals of the study were to 1) determine a Landsat-derived index which would allow us to estimate pre-fire spruce beetle severity using a multi-date image difference, and 2) determine the relationship of NDVI two years after the burn to pre-fire beetle outbreak severity, accounting for the influences of topography, weather at time of burning, and pre-disturbance NDVI.

## Methods

### Study area

**Fig 1:**
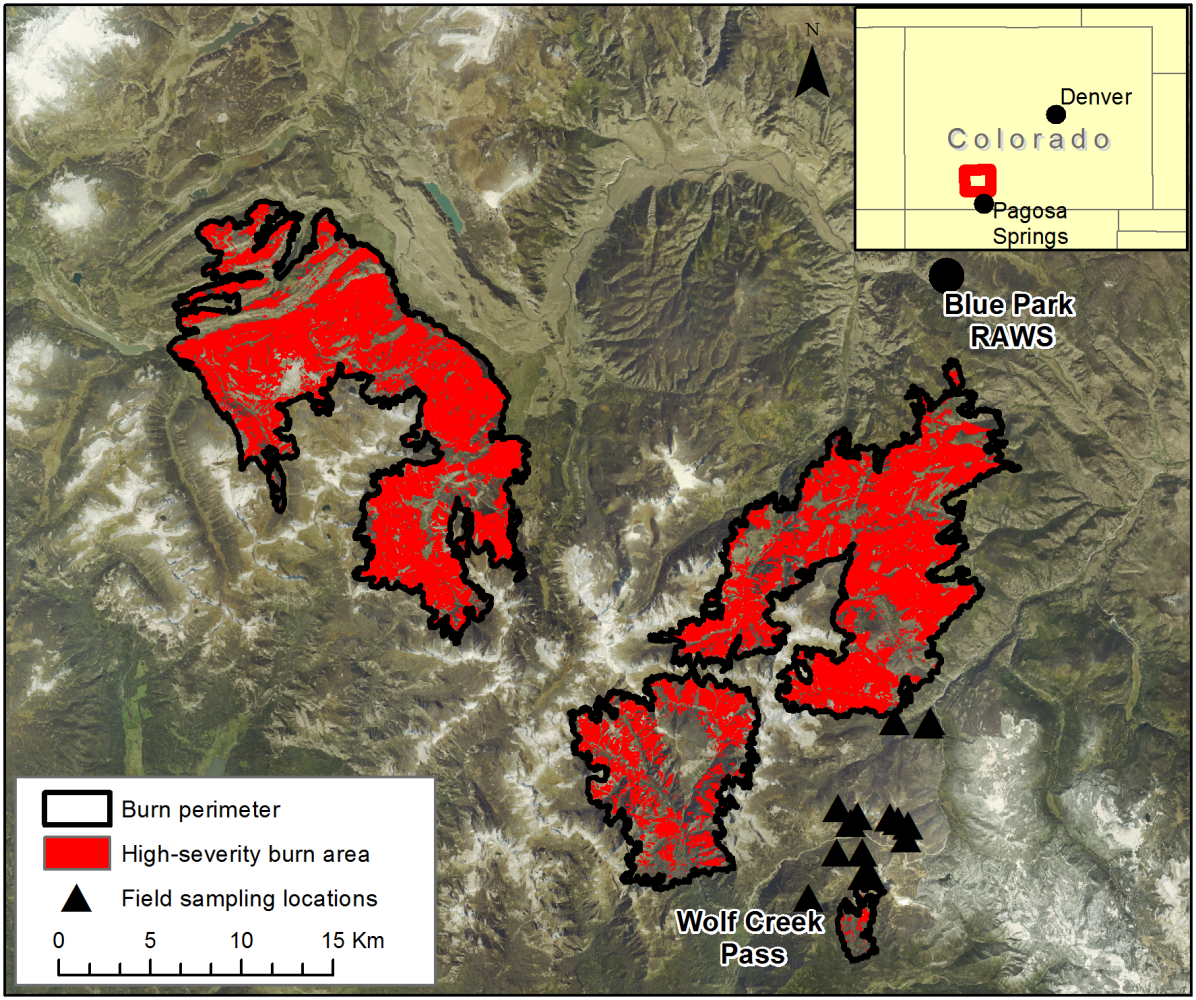
Overview map of the West Fork Complex burn area. Locations of the nearest weather station (Blue Park RAWS) and field sampling locations around Wolf Creek Pass are indicated. Area in red indicates burn area classified as ‘high severity’ by the US Forest Service Burned Area Emergency Response (BAER). Red square in inset shows the location of the study area within Colorado. Base imagery is from the USGS National Map server.

The West Fork Complex fire burned from June 5 –July 6, 2013. The complex consisted of three lightning-caused wildfires: Papoose (20,084 ha), West Fork (23,705 ha), and Windy Pass (573 ha). A total of over 44,000 hectares of subalpine spruce/fir forest in the Rio Grande National Forest, San Juan National Forest, and private lands northeast of Pagosa Springs, Colorado, were burned (Fig 1). Fire spread was driven by strong winds and high temperatures, causing up to 7,500 ha of spread in a single day. Firefighting management was minimal due to steep terrain and hazardous conditions presented by fire behavior in beetle-killed forest, and because the fire primarily burned areas designated as wilderness. The US Forest Service’s Burned Area Emergency Response (BAER) program classified the majority of the burn as ‘high-severity’, indicating complete canopy mortality and loss of understory vegetation [57].

The burn area was dominated by *P*. *engelmannii* and *A*. *lasiocarpa*, with some lodgepole pine (*Pinus contorta)*, quaking aspen (*Populus tremuloides*), and Douglas fir (*Pseudotsuga menziesii*). Elevation ranges from ~2700–4000 m a.s.l. with steep slopes and rugged topography. Mean temperatures range from -7.80°C in January to 11.50°C in July, and annual precipitation is 95 cm (PRISM climate data; http://www.prism.oregonstate.edu/). Significant spruce beetle activity within the burn perimeter was first detected by ADS in 2004 (data available from US Forest Service; https://www.fs.usda.gov/detail/r2/forest-grasslandhealth/?cid=fsbdev3_041629). The outbreak was very severe, affecting more than 80% of spruce/fir forest within the study area by the time of the 2013 fire.

### Landsat image processing

**Fig 2:**
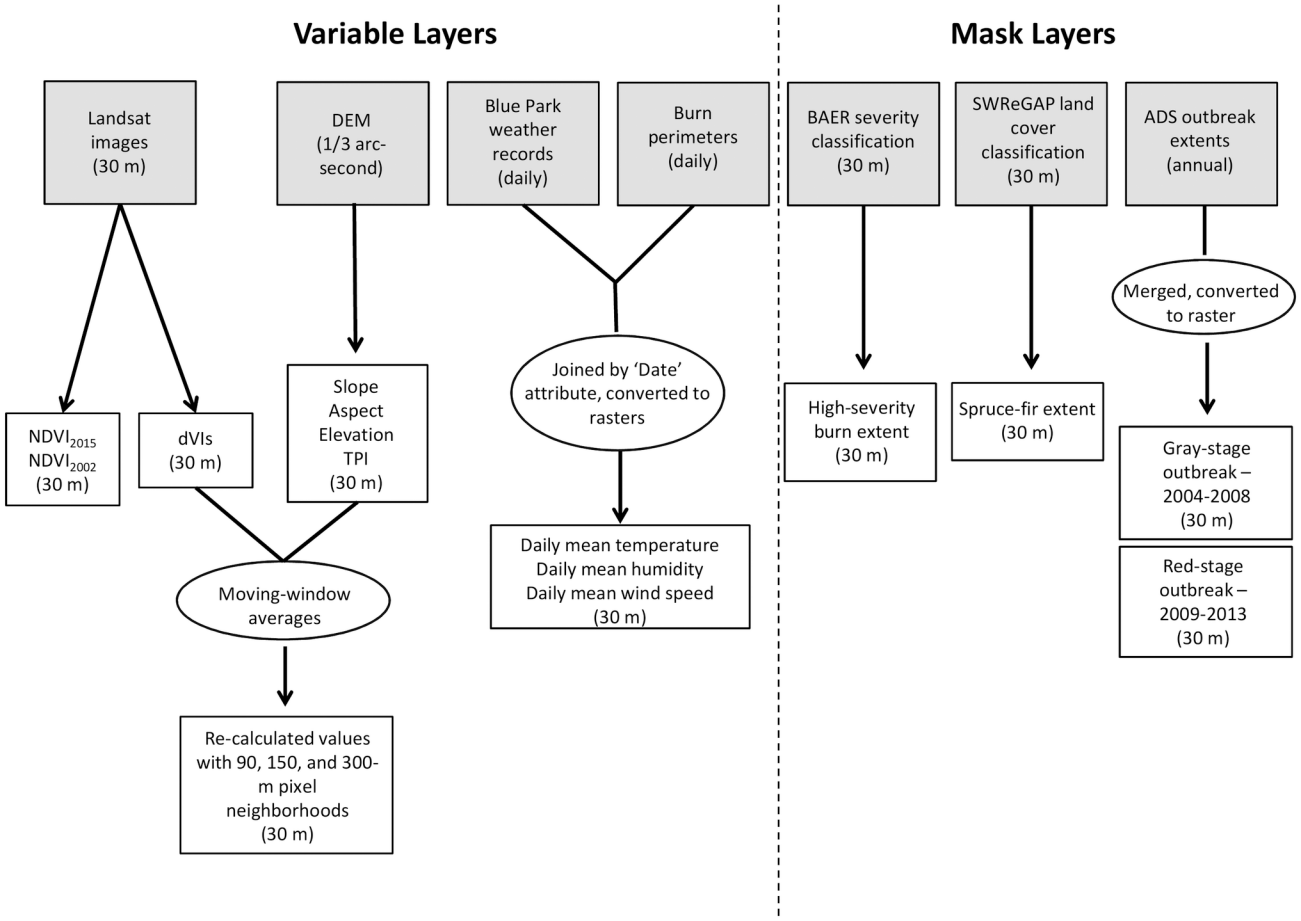
Processing steps used to derive model variable layers from data sources.

Processing steps for Landsat images and other explanatory variables are outlined in Fig 2. We acquired Landsat 7 ETM+ and Landsat 8 OLI (path 34, row 34) surface reflectance images collected in 2002, 2006, 2012, 2013, and 2015 (see Table 1 for image dates and sensor types). Images were pre-processed to surface reflectance using the Landsat Ecosystem Disturbance Adaptive Processing System (LEDAPS) [58]. The 2002 image predated the earliest detection of spruce beetle mortality by ADS, and was assumed to represent undisturbed canopy conditions. Subsequent images represent distinct points in the disturbance history of the site: mid-beetle outbreak (2006), mid-beetle outbreak and immediately pre-fire (2012), immediately post-fire (2013), and following two years of post-fire recovery (2015). We selected cloud-free images representing growing-season conditions at each time point (August, or the latest available growing-season date for which a cloud-free image was available). The 2006 and 2012 images contained missing data areas due to Landsat 7’s Scan Line Corrector Error, which accounted for ~5% of the study area. We excluded these missing data areas in the 2012 image from the final analysis.

**Table 1:** Date and sensor type for Landsat scenes used in analysis.

Image	Sensor	Date
Pre-disturbance	L7 ETM+	August 10, 2002
Mid-beetle outbreak	L7 ETM+	June 18, 2006
Post-beetle outbreak, pre-fire	L7 ETM+	June 18, 2012
Immediately post-fire	L8 OLI	August 16, 2013
2 years post-fire	L8 OLI	August 06, 2015

Although the images were pre-processed to eliminate atmospheric biases between image dates, slight band differences between Landsat 7 ETM+ and Landsat 8 OLI may result in systematic biases between images collected with the different sensor types. In order to eliminate this bias when comparing images, we applied a normalization technique to the 2015 image using pseudo-invariant features (PIFs). We manually selected 40 PIFs as single pixels representing non-vegetative features where reflectance should be constant between image dates (such as bare soil above tree line, water bodies, and major roads). A linear regression calculation between each image band was used to adjust the 2015 image according to the method described by Schott et al. [59]. In all cases the fit of the regression line used in band adjustment calculations was R^2^ ≥ 0.86.

### Beetle severity indices

#### Field validation

We assessed the ability of Landsat-derived VIs to approximate spruce beetle severity using field measurements of beetle-caused spruce mortality. In August of 2015, we collected measurements in 58 unburned, beetle-affected spruce/fir plots within ~5 km of the West Fork Complex burn perimeter (see Fig 1). Fifteen sampling locations were chosen in ArcMap 10.0 [60] to achieve a diverse representation of topographic characteristics, outbreak severities, and outbreak ages (determined by ADS). Including plots in different outbreak stages accounted for potential differences in spectral response caused by regeneration in older beetle-killed stands. Each sampling location consisted of a 180 m-long east-west transect with four 20 x 20 m evenly spaced sampling plots. In one transect we only established two plots, because spruce stands were surrounded by flat, wet subalpine fir-dominated site conditions which are uncharacteristic of the total study area. Outbreak severities ranged from 0–100% beetle-caused mortality in overstory trees.

We used a handheld GPS to place plots within ~3 m of the center of a 2 x 2 Landsat pixel grid (60 x 60 m). We measured diameter at breast height (DBH) of all dead *P*. *engelmannii* trees with evidence of recent beetle activity within each 20 x 20 m plot area, and converted these measurements to total basal area. Basal area of beetle-killed trees within plot areas (400 m^2^) was our selected metric of beetle outbreak severity, and was assumed to estimate total change in canopy cover from pre-outbreak to post-outbreak. Our metric of beetle severity is therefore an absolute value of beetle-killed *P*. *engelmannii* basal area per 400 m^2^ (20 x 20 m plot area) rather than a percentage of total canopy. Standing dead trees with no evidence of beetle activity were small in diameter, and we assumed that these trees did not significantly affect the spectral changes resulting from beetle outbreak.

#### Vegetation indices

We tested seven VIs which have been shown to respond to canopy disturbance: the Normalized Difference Moisture Index (NDMI) [35,61], Normalized Burn Ratio (NBR) [25,62], Vegetation Condition Index (VCI) [30,63], Moisture Stress Index (MSI) [30,64], and two Disturbance Indices (DI and DI’) based on the Tasseled Cap transformation [29,65–67]. VIs were calculated using combinations of two or more Landsat bands (see Table 2 for index calculations).

**Table 2:** Equations used to calculate Landsat vegetation indices (VIs) used to approximate beetle severity.

Index	Equation
NDVI	(Near-infrared–Red)/(Near-infrared + Red)
NDMI	(Near-infrared–Mid-infrared)/(Near-infrared + Mid-infrared)
NBR	(Near-infrared–Thermal-infrared)/(Near-infrared + Thermal-infrared)
VCI	Thermal-infrared/Near-infrared
MSI	Mid-infrared/Near-infrared
DI	TCBright–(TCGreen + TCWet)*
DI’	TCWet—TCBright*

*Refers to Tasseled Cap Brightness, Greenness, and Wetness transformations of Landsat bands, rescaled according to the method described by Healey et al. [67].

For each VI, we calculated a multi-date image difference by subtracting 2002 pre-disturbance values from the 2015 value (dVI = VI2015 –VI_2002_). We compared these image differences to field measurements of beetle-caused overstory mortality by calculating the means of dVI values extracted from the 2 x 2 (60 x 60 m) pixel grid area surrounding field plot centers. We used mean values to account for potential spatial inaccuracies in the GPS location of the plot and overlay with the Landsat grid. Relationships between dVIs and plot-level values of basal area of beetle-killed trees were assessed using ordinary least squares (OLS) regression. The dVI which yielded the highest OLS R^2^ value was assumed to be the best indicator of beetle outbreak severity, and the difference in the selected index from 2002 to 2012 (VI_2012_ –VI_2002_) was included as an explanatory variable in post-fire NDVI models.

### Other explanatory variables

#### Topography

Topographic variables included slope, elevation, aspect, and topographic position index (TPI). TPI is a numeric indicator of slope position, with higher values representing locations closer to ridgetops and lower values representing valley bottoms [68]. Aspect was transformed to relative ‘northness’ using the formula abs(aspect– 180), so that values range from 0–180 as aspect increases from south-facing to north-facing. All topographic predictor variables were derived from a 1/3 arc-second digital elevation model (DEM), resampled to a resolution of 30 m.

#### Weather

NDVI models included variables accounting for daily weather conditions over the two-week burn period. This was done using daily burn perimeter maps, which we obtained from the USGS Geospatial Multi-agency Coordination (GeoMAC) Wildland Fire Support service [69]. Each of these daily burn areas was classified with the corresponding mean daily values for air temperature, humidity, and wind speed. Daily weather station data was obtained from the Blue Park Remote Automated Weather Station (RAWS) [70].

#### Outbreak stage

**Fig 3:**
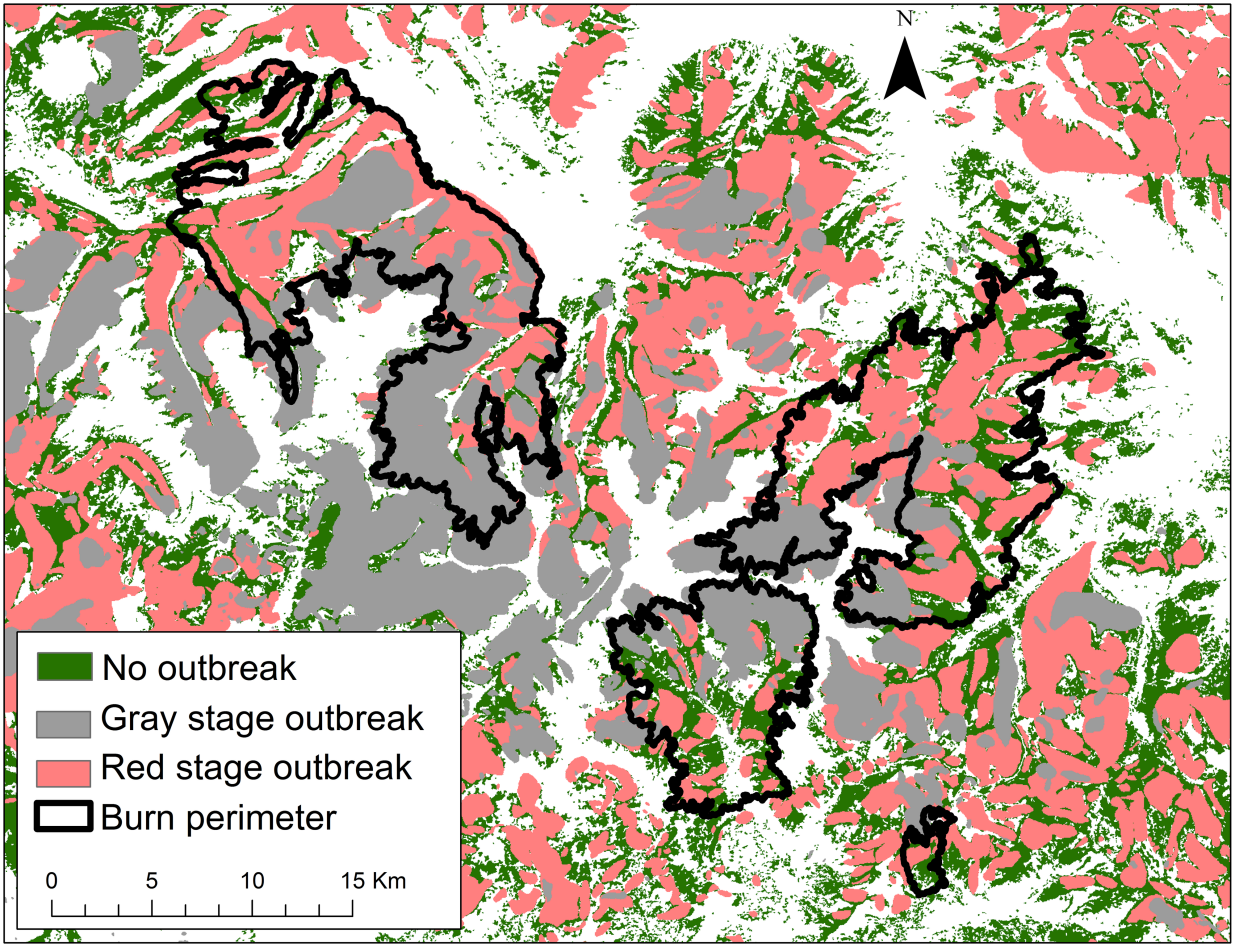
Extent of study area classified as red or gray-stage beetle outbreak in 2012. Green area depicts spruce/fir forest with no outbreak detected.

We determined outbreak stage using the earliest year of spruce beetle detection from ADS data. Using annual ADS extents for all years since 1994, we determined that 2004 was the earliest year when significant spruce beetle activity was mapped within the study area. Polygon areas with detection years 2004–2008 were classified as gray-stage, while polygons with detection years 2009–2013 were classified as red-stage (Fig 3). These areal extent layers were used to clip explanatory variable areas to red and gray-stage locations. We examined red and gray-stage locations in separate models to determine how relationships between outbreak severity and vegetation recovery varied between outbreak stages.

### NDVI models

Explanatory variable layers were clipped to areas of spruce/fir forest cover type which burned at high severity. The spruce/fir forest cover mask layer was derived from the Southwest Regional Gap Assessment (SWReGAP) land cover classification [71], using category labels which corresponded to subalpine spruce/fir forest type (Fig 3). The high severity mask layer was derived from a 4-level BAER classification product based on the Landsat-derived relativized change in Normalized Difference Burn Ratio (RdNBR; Fig 1). We generated a predictor variable matrix by sampling pixel values from clipped layers along a 60x60 m point lattice (sampling at every other pixel).

#### Sequential autoregression

The effects of spatial autocorrelation must be taken into account when assessing patterns in a contagious disturbance such as wildfire, because spatial dependence in either the response or the explanatory variables can violate assumptions of observation independence and inflate parameter values [72,73]. Variogram analysis revealed spatial autocorrelation in the NDVI layers for up to ~500 m of lag distance. We therefore modeled post-fire vegetation cover using spatial error sequential autoregressive models (SAR) to account for the effects of positive spatial autocorrelation in the data. The formula for the error SAR model is given by the following equation:

Y=Xβ+ λW(Y−Xβ)+ ε

where Y is the dependent variable, X is a given predictor, β is the parameter estimate for X, λ is the autoregressive coefficient, W is a spatial weights matrix, and ε is a random error term. We determined the spatial weights matrix W using the inverse distance of neighbors within 125 m of sample locations. All statistical analyses were carried out in R 3.3.1 [74] using the ‘spdep’ package [75].

#### Variable scale

To further account for the spatially connected nature of wildfire, we considered how variable scale may affect relationships between vegetation recovery patterns and explanatory variables. Wildfire is a rapid-spreading, contagious process, and fire behavior is likely to be influenced by topographic and fuel characteristics over a broader area than that covered by a 30-m Landsat pixel. Because fire behavior influences the degree of fuel consumption and burning intensity across the landscape, and can ultimately influence patterns of vegetation recovery [76], we expect that spatial neighborhood effects influence the relationship of topographic and fuel variables to post-recovery NDVI assessed at 30-m resolution. We accounted for neighborhood effects of explanatory variables using square moving-window average functions on our topographic variables and beetle severity index, which implicitly accounts for fuel structure. This process generated new 30-m raster layers by calculating new values for each pixel using the averages of surrounding pixels within our selected window sizes of 90, 150, and 300 m (corresponding to 3x3, 5x5, and 10x10 pixel grids, respectively). We determined the most appropriate scale of analysis for each variable using univariate SAR models for each variable at each scale to predict NDVI_2015_. The best-fitting model based on Akaike’s Information Criterion (AIC) value was used to select the scale for each variable to be included in the final multivariate model. This scale selection process ensures that the explanatory power of each variable is maximized in the final multivariate model [77,78].

#### Multivariate models

After determining the best-fitting scales for explanatory variables, we used a stepwise selection procedure to select a model from a full set of explanatory variables. This full model can be described by the following equation:

$$NDVI_{2015}\sim \;dVI+slope+northness+elevation+TPI+mean\;air\;temp.\;+\;mean\;humidity+mean\;wind\;speed+NDVI_{2002}$$

We selected the combination of variables which minimized AIC value for both the red and gray stage. Relative importance of each variable to the final model was determined by removing variables from the final selected model and calculating the change in AIC (ΔAIC).

## Results

### Beetle severity indices

**Fig 4:**
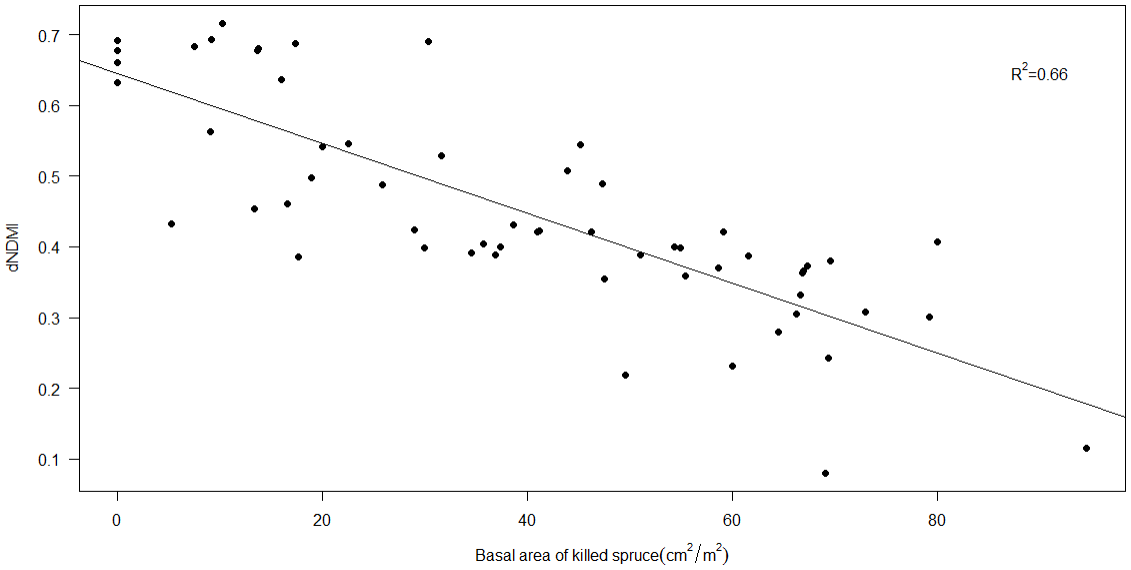
Relationship of measured basal area of killed *P*. *engelmannii* in field plots to dNDMI values at plot locations. Plot areas are 20 x 20 m.

**Fig 5:**
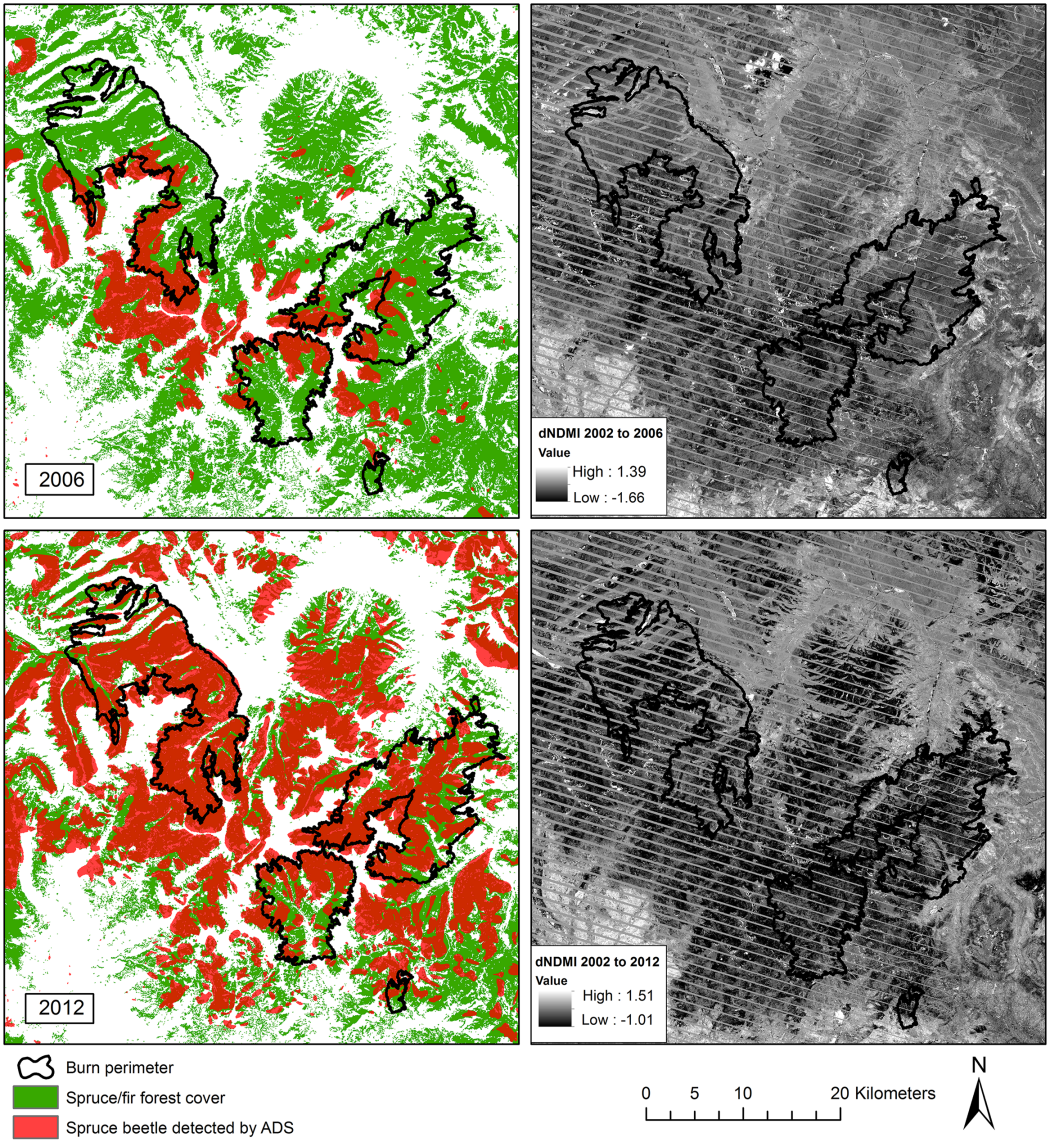
Comparison between spruce beetle outbreak extent detected by ADS from 2002 to the indicated year (left; ADS polygons shown in orange) and dNDMI (right). Top dNDMI = NDMI_2006_ –NDMI_2002_; bottom dNDMI = NDMI_2012_ –NDMI_2002_. Color scales for dNDMI are based on standard deviations within images.

R^2^ values indicated that dNDMI was the index most strongly correlated to field-measured basal area of beetle-killed spruce (Table 3). The R^2^ value of the OLS regression was 0.66, indicating a relatively strong correlation (Fig 4). Furthermore, visual inspection of spatial patterns in dNDMI at multiple time points showed that values were responsive to outbreaks detected by ADS (Fig 5). Although the magnitude of dNDMI values varies as a result of scale differences in the post-outbreak image, there are clear spatial patterns within images indicating that lower values of dNDMI (darker-colored areas in the right-hand column of Fig 5) correspond to known beetle outbreaks (areas detected by ADS; shaded orange in the left-hand column of Fig 5) at multiple time points. The close relationship between dNDMI and field-measured spruce mortality, in addition to temporal trends of ADS detection, indicates that dNDMI is a good proxy for beetle outbreak severity. dNDMI was therefore selected as a proxy for beetle outbreak severity in NDVI models.

**Table 3:** R^2^ values from OLS regression tests comparing 2002–2015 dVIs to the beetle-killed basal area of spruce in field plots measured in 2015.

Index	R^2^
dNDMI	0.66
dDI	0.65
dVCI	0.62
dNBR	0.61
dNDVI	0.60
dMSI	0.60
dDI’	0.56

### NDVI recovery

**Fig 6:**
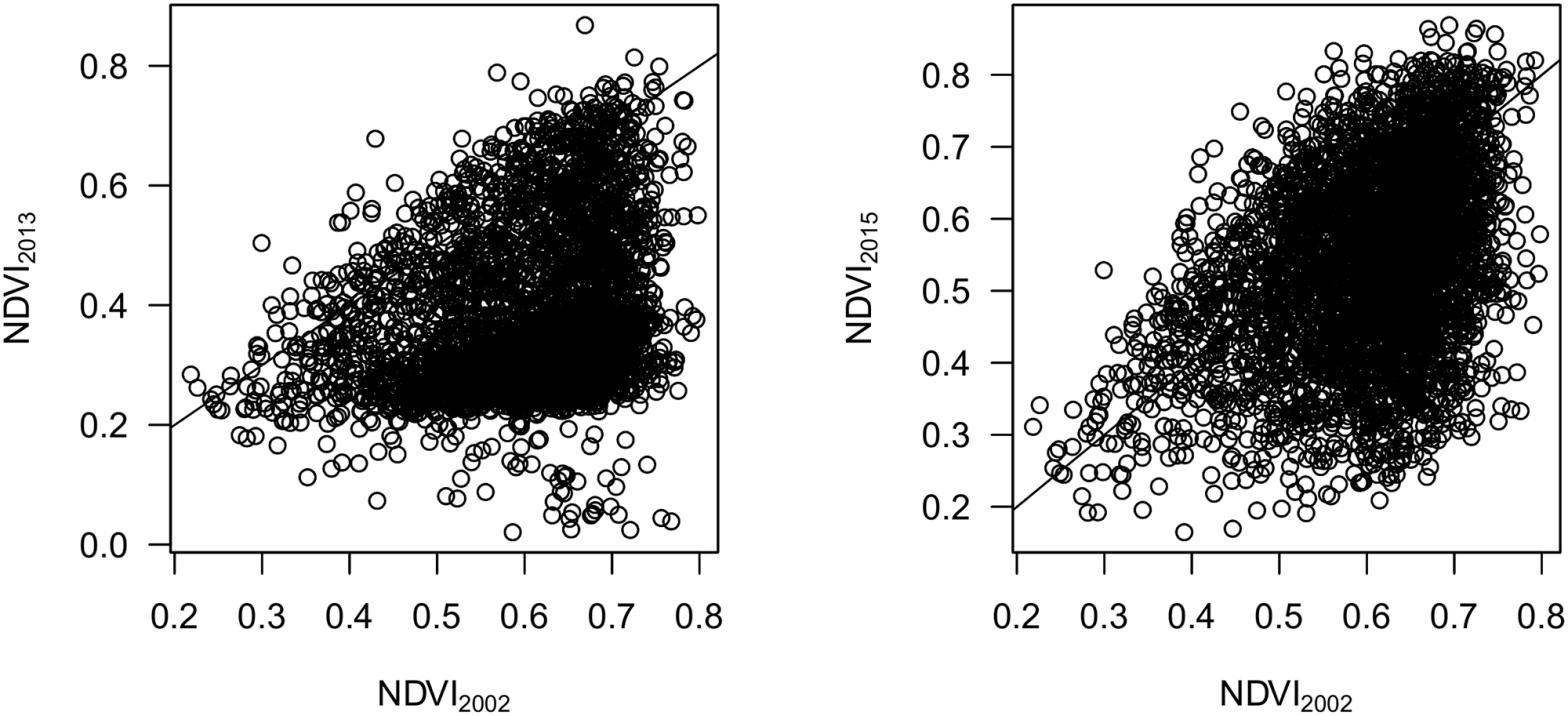
Pattern in NDVI change for locations which burned at high severity compared to undisturbed 2002 conditions. Solid line represents a 1:1 relationship.

**Fig 7:**
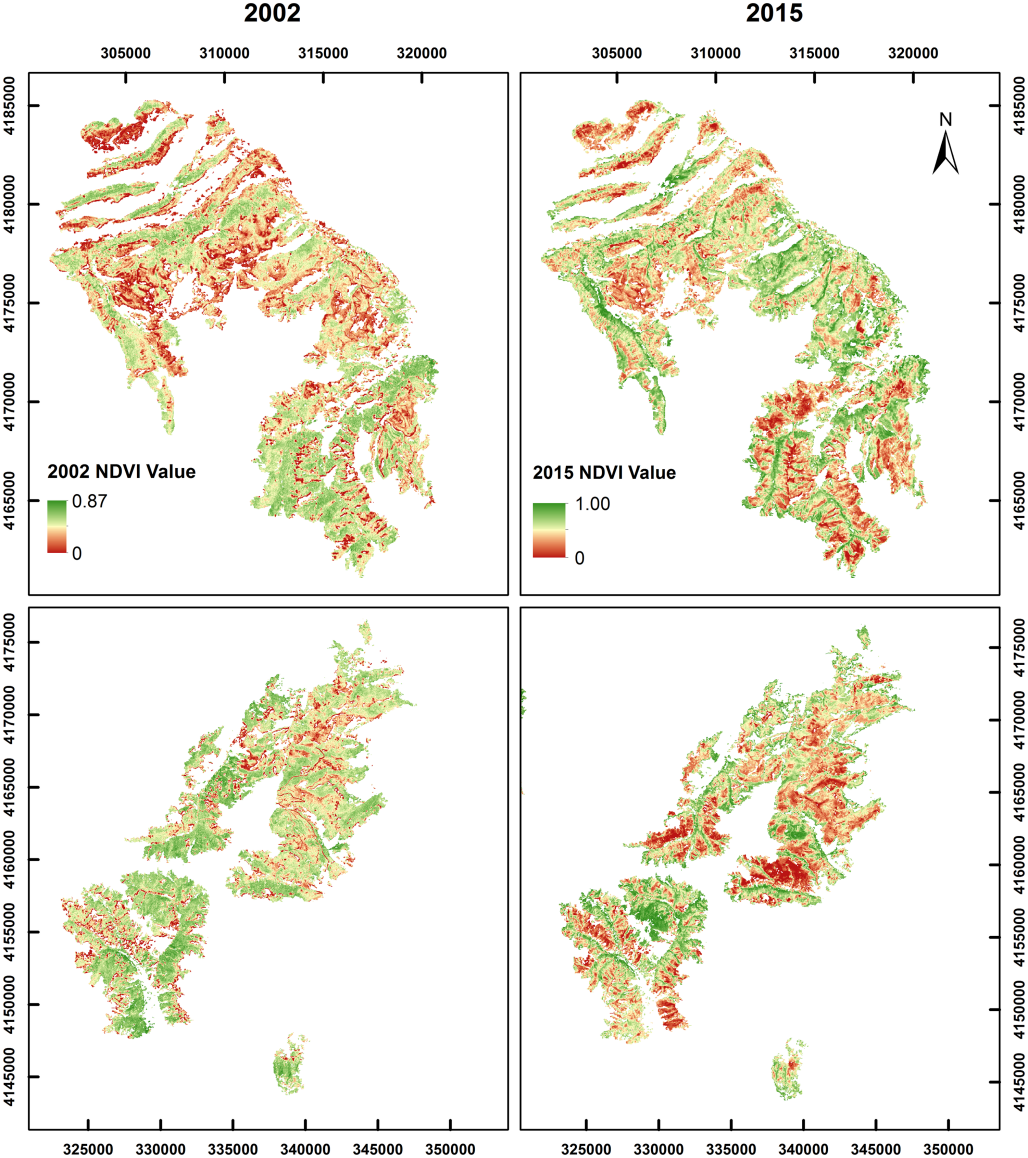
Growing-season NDVI for the Papoose burn area (top row), and West Fork and Windy Pass burn areas (bottom row). NDVI images are clipped to spruce/fir forest cover type. Left images are from 2002 (pre-disturbance) and right images are from 2015 (2 years post-fire recovery).

Fig 6 illustrates the pattern of NDVI recovery from 2013 (immediately after the fire) to 2015 for a randomly selected subset of NDVI pixels. Comparison of NDVI_2013_ and NDVI_2015_ to NDVI_2002_ reveals that NDVI has increased toward pre-disturbance values in the two years following wildfire, compared to relatively homogenous values in 2013. However, NDVI_2015_ values are generally lower than their corresponding 2002 values. The pattern of recovery is heterogeneous, with some areas in the southern portions of the West Fork and Papoose burn areas showing slower recovery compared to the rest of the burn area (Fig 7).

### SAR model results

#### Univariate

Univariate relationships between explanatory variables and NDVI_2015_ determined by SAR are summarized in Table 4. These results present the best-fitting scales for all variables where a moving-window average calculation was applied. The relationship of vegetation cover to dNDMI is highly significant (p < 0.0001) in both the red-stage and gray-stage sample subset, but the slopes of the relationships are opposite. dNDMI is positively correlated to NDVI_2015_ in red-stage models, indicating that lower values of dNDMI (indicating greater mortality from spruce beetle outbreak) are correlated with lower vegetation cover in 2015. This relationship is negative in gray-stage models.

**Table 4:** Results of univariate SAR models predicting 2015 NDVI for point locations in gray-stage and red-stage pre-fire spruce beetle outbreak, with the best-performing scale of moving-window averages selected for dNDMI, slope, northness, and TPI. Variables in bold are significant within a 95% confidence interval. Models are ranked by AIC value.

Gray-stage: Variable	β	Std. error	AIC	p
**NDVI**_**2002**_	**0.48**	**8.9 x 10**^**−3**^	**-51473**	**<0.0001**
**northness150**	**6.9 x 10**^**−4**^	**3.7 x 10**^**−5**^	**-49056**	**<0.0001**
**elevation**	**-3.0 x 10**^**−4**^	**1.6 x 10**^**−5**^	**-49047**	**<0.0001**
**slope300**	**-3.7 x 10**^**−3**^	**3.3 x 10**^**−4**^	**-48844**	**<0.0001**
**TPI300**	**-0.16**	**0.033**	**-48738**	**<0.0001**
**dNDMI90**	**-0.068**	**0.015**	**-48736**	**<0.0001**
**air temperature**	**2.6 x 10**^**−3**^	**1.0 x 10**^**−3**^	**-48723**	**<0.05**
humidity	-2.5 x 10^−4^	1.8 x 10^−4^	-48718	0.16
wind speed	5.8 x 10^−4^	2.5 x 10^−3^	-48716	0.82
Red-stage: Variable	β	Std. error	AIC	p
**2002 NDVI**	**0.49**	**7.8 x 10**^**−3**^	**-59878**	**<0.0001**
**elevation**	**-2.3 x 10**^**−4**^	**1.1 x 10**^**−5**^	**-56696**	**<0.0001**
**northness150**	**3.5 x 10**^**−4**^	**3.3 x 10**^**−5**^	**-56410**	**<0.0001**
**TPI300**	**-0.27**	**0.028**	**-56397**	**<0.0001**
**dNDMI300**	**0.24**	**0.028**	**-56373**	**<0.0001**
**slope150**	**-7.7 x 10**^**−4**^	**1.6 x 10**^**−4**^	**-56323**	**<0.0001**
**humidity**	**5.0 x 10**^**−4**^	**1.9 x 10**^**−4**^	**-56306**	**<0.01**
air temperature	8.6 x 10^−4^	7.5 x 10^−4^	-56301	0.25
wind speed	-1.2 x 10^−3^	2.1 x 10^−3^	-56300	0.56

NDVI_2002_ is the strongest single-variable predictor in both red and gray-stage models, based on AIC value. All topographic variables are significant in both subsets, while weather variables are not consistently significant. Topographic variables were selected at greater spatial scales, either at 150 m or 300 m. In the gray-stage subset, all topographic variables predicted NDVI_2015_ more accurately than dNDMI. In the red-stage subset, dNDMI was a more accurate predictor than either slope or TPI, or any weather variable.

#### Multivariate

**Fig 8:**
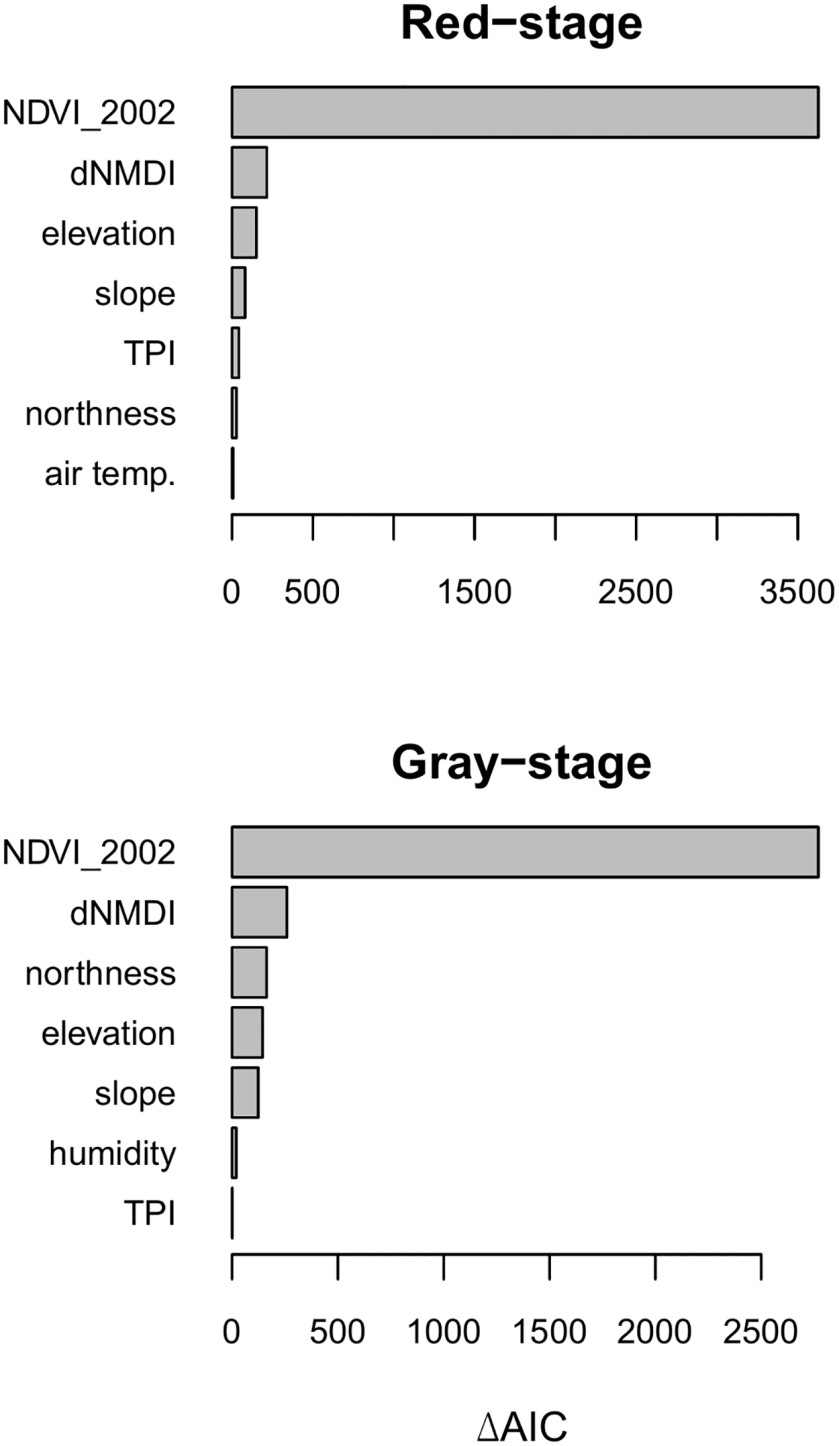
Variable importance plots for the best-fitting multivariate models for red and gray-stage outbreak locations. Variable importance is determined by the ΔAIC between the full model and model with the indicated variable removed.

In both gray-stage and red-stage models, including dNDMI as a predictor improved model fit according to AIC. The best-fitting models selected from a full set of variables are given in Table 5. The best-fitting model for gray-stage locations explained 71% of variance in NDVI_2015_, and included all explanatory variables except air temperature and wind speed. The top-performing model for red-stage locations explained 68% of variance in NDVI_2015_ and included all explanatory variables except humidity and wind speed. Variable importance calculations showed that NDVI_2002_ is by far the most important variable in both red and gray-stage models (Fig 8). dNDMI had a higher importance value in both models than any other explanatory variables.

**Table 5:** Top-performing multivariate SAR models predicting 2015 NDVI for point locations in red-stage and gray-stage of spruce beetle outbreak prior to fire.

Stage	Best-fitting model	R^2^
Gray-stage	NDVI_2015_ ~ NDVI_2002_ + dNDMI90 + slope300 + northness150 + TPI300 + elevation + humidity	0.71
Red-stage	NDVI_2002_ + dNDMI300 + slope150 + northness150 + TPI300 + elevation + air temp.	0.68

Parameter estimates for the top-performing multivariate SAR models revealed variable relationships similar to those determined by univariate models (Table 6). dNDMI is highly significant (p <0.0001) and exhibits a positive relationship to NDVI_2015_ in both gray-stage and red-stage models, but NDVI_2002_ is the strongest predictor in both model subsets. Topographic variables are also significant predictors in all models, with slope, elevation, and TPI exhibiting negative correlations with NDVI_2015_ while northness exhibits a positive correlation. Humidity is negatively correlated with NDVI_2015_ in the gray-stage model while air temperature is negatively correlated with NDVI_2015_ in the red-stage model. All signs of variable relationships are consistent between gray-stage and red-stage models. The parameter estimate for dNDMI is greater in magnitude in the top-performing red-stage model (β = 0.40 ± 0.027) than in the top-performing gray-stage model (β = 0.25 ± 0.015), indicating that dNDMI has a greater influence on NDVI_2015_ in the red-stage model.

**Table 6:** Parameter estimates, standard errors, and significance values for top-performing multivariate SAR models predicting 2015 NDVI. Variables in bold are significant within a 95% confidence interval.

Gray-stage: Variable	β	Std. Error	p-value
**dNDMI90**	**0.25**	**0.015**	**<0.0001**
**NDVI**_**2002**_	**0.52**	**9.5 x 10**^**−3**^	**<0.0001**
**slope300**	**-3.4 x 10**^**−3**^	**3.0 x 10**^**−4**^	**<0.0001**
**northness150**	**4.5 x 10**^**−4**^	**3.5 x 10**^**−5**^	**<0.0001**
**elevation**	**-1.9 x 10**^**−4**^	**1.6 x 10**^**−5**^	**<0.0001**
**TPI300**	**-0.065**	**0.031**	**<0.05**
**humidity**	**-7.4 x 10**^**−4**^	**1.6 x 10**^**−4**^	**<0.0001**
Red-stage: Variable	β	Std.Error	p-value
**dNDMI300**	**0.40**	**0.027**	**<0.0001**
**NDVI**_**2002**_	**0.49**	**7.9 x 10**^**−3**^	**<0.0001**
**slope150**	**-1.3 x 10**^**−3**^	**1.4 x 10**^**−4**^	**<0.0001**
**northness150**	**1.8 x 10**^**−4**^	**3.3 x 10**^**−5**^	**<0.0001**
**elevation**	**-1.4 x 10**^**−4**^	**1.2 x 10**^**−5**^	**<0.0001**
**TPI300**	**-0.17**	**0.026**	**<0.0001**
**air temperature**	**-1.9 x 10**^**−3**^	**6.6 x 10**^**−4**^	**<0.01**

## Discussion

Because dNDMI is negatively correlated with spruce beetle outbreak severity, the results of multivariate SAR models indicate that recovery of NDVI in the West Fork Complex fire was negatively correlated with the severity of spruce beetle outbreaks which occurred in the decade or so prior to the fire. The direction of the univariate relationship between dNDMI and NDVI_2015_ matched that of the multivariate relationship in red-stage models but was reversed in gray-stage models, possibly indicating that the compounded disturbance effect becomes less significant with increasing time between disturbances. However, when all relevant variables were accounted for there was a consistently negative relationship between beetle outbreak severity and NDVI_2015_ in both stages. Although dNDMI was a less significant predictor of NDVI_2015_ than NDVI_2002_ (according to ΔAIC), the significant correlation between the indicator of beetle severity and NDVI_2015_ suggests the presence of a compounded disturbance effect on the rate and trajectory of vegetation recovery.

Our results add a new dimension of understanding to those of recent studies which have found no correlation between outbreak severity and subsequent fire severity when accounting for differences in outbreak stage, burning conditions, or topography [46]. Most of these studies have assessed fire severity by measuring immediate post-fire impacts to aboveground vegetation, using remotely sensed indices such as dNBR or RdNBR [43,48,50–52] or field-based metrics such as scorch height, percent surface char, or percent overstory mortality [39,48,50]. We focused on the effects of high-severity fire only, which made up a majority of the West Fork Complex burn area. Previous studies have addressed whether there is a linked interaction between beetle outbreaks and the impact of fire on existing vegetation, but may not fully address all mechanisms of compounded interactions on vegetation recovery (see Buma [4] for a review of linked and compound disturbance). We propose that a significant negative relationship between beetle outbreak severity and vegetation recovery was observed in the West Fork Complex because the pre-fire beetle outbreak may have played a significant role in fire behavior at the soil surface; an effect which has not been thoroughly explored by previous beetle-wildfire interaction studies.

This difference in linked vs. compounded disturbance effects can be seen when comparing the results of our study to the findings of Andrus et al. [48], which also examined beetle-wildfire interactions in the West Fork Complex. That study found no effect of spruce beetle outbreak on canopy tree mortality, percent surface char, or RdNBR immediately after the fire. Although those results provide important insights into the effect of spruce beetle outbreaks on fire behavior and canopy mortality, these metrics of fire severity may not account for ecologically important impacts to chemical properties of soils, vegetative seed banks, or resprouting roots. Moreover, remotely sensed metrics based on differences between pre-fire and post-fire imagery may underestimate fire severity if greenness in the pre-fire imagery is reduced by a severe beetle outbreak [78]. This may be a reason why previous studies have found a consistently negative correlation between bark beetle outbreaks and RdNBR in subsequent fires [51,52].

Effects of spruce beetle outbreak on regeneration processes may be a more significant ecological impact than effects to canopy loss, due to the typical high severity of fires in subalpine forests. High canopy mortality is expected in subalpine systems because climatic conditions typically make fires infrequent, and the long interval between fire results in dense fuel stocking [21]. *P*. *engelmannii* and *A*. *lasiocarpa* are shade-tolerant species, meaning that mature stands become stocked with ladder fuels which incur a high probability of active crown fire [23]. They are also thin-barked species, and mortality can be high from low-intensity surface fire alone [22,79]. Because we expect subalpine fires to be stand-replacing regardless of beetle-caused changes to canopy structure [22,80], it is important to consider other mechanisms by which multiple disturbances may interact to produce compounded effects. If beetle outbreaks are significantly increasing surface fuel loads, this may explain impacts on vegetation recovery resulting from increased heat released by burning at the soil surface [81].

### dNDMI as an indicator of beetle severity

Past studies have assessed pre-fire beetle mortality in the field after fire has occurred, which requires close examination of all trees within a field plot for larval galleries beneath the bark. This is a time-consuming process, and may also be prone to underestimation of mortality when the bark and wood surface have been damaged by fire [28]. Differencing and single-date classification of NDMI time series have proven to be effective methods for detecting and quantifying outbreaks of North American and European spruce beetle [29–31], mountain pine beetle [32,33], and canopy gaps due to disturbance in coniferous forests [34]. In our study, dNDMI was a reliable estimator of spruce mortality from bark beetles, and other dVIs also correlated well with field measurements. Remote sensing estimates likely provide a more objective measurement of pre-fire beetle disturbance compared to field measurements taken after fires have occurred.

We observed that spruce cover was high in most of our study area and that high abundance of subalpine fir was restricted to flat valley bottoms, which made up a low proportion of the total area. Our severity quantification method was therefore focused on spruce-dominated stands (where spruce made up >50% of total basal area). Consideration should be taken in applying the dNDMI severity quantification method to areas with more mixed forest communities, as it is possible that growth in secondary species between image dates could cause dNDMI to underestimate spruce mortality [36]. In our study area these areas included stands classified as aspen woodlands, which represented ~8% of the total burn area and were not included in models.

### NDVI and vegetation recovery

Previous wildfire recovery studies have shown that NDVI tends to increase rapidly in the two years following fire occurrence [44,45]. NDVI in the West Fork Complex also increased rapidly, and overall NDVI values are correlated with pre-disturbance values. Post-fire vegetation is characterized by grass and forb understory rather than by the pre-fire forest canopy, but because NDVI is sensitive to understory vegetation [82], the importance of NDVI_2002_ in models of NDVI_2015_ indicates that some factors relating to site greenness are unaltered by fire. Differences between pre-disturbance and post-disturbance NDVI may be the result of alteration of soils and microclimate which affect the ability of understory species to re-establish.

It is important to note that understory recovery is not necessarily an indicator of overstory regeneration [82]. However, rates of understory succession have been shown to affect forest seedling regrowth, community resilience, and recovery of soil properties. For example, reduced cover following high-severity wildfire in subalpine forests has been found to correlate with reduced soil nitrogen, which could have long-term impacts on seedling establishment [83]. Reduced recruitment of early successional species can also be an indicator of severely altered soil properties following fire [84]. Given the high severity of the West Fork Complex, it is likely that altering of soil properties will influence variation in overall vegetation recovery across the burn area. However, future differences in vegetation composition will be also determined by climate, seed dispersal, topography, and future disturbance [38].

### Mediating factors in the relationship between beetle severity and vegetation recovery

Comparing univariate to multivariate relationships between beetle outbreak severity and NDVI_2015_ in red and gray stages reveals that in the gray stage, the effect of beetle severity is mediated to a greater extent by other explanatory variables. This difference may indicate that the effect of beetle-caused canopy mortality on fire impacts diminishes over time. This may be due to the fact that fine surface fuels decompose or are lost from the site after the initial outbreak [10]. Canopy loss from spruce beetle outbreak also allows for the recruitment of grass, forbs, and shrubs in the understory, which may be able to germinate or resprout rapidly after the fire [10,85].

Multivariate models indicated that outbreak severity has a significant influence on post-fire recovery, but did not have a greater effect than topography or pre-disturbance NDVI. Topography is important in influencing fire behavior and micro-climate conditions which can promote or impede vegetation recovery [51,86]. The influence of topographic variables in multivariate models was expected, given results of previous NDVI recovery studies [44,45]. The selection of topographic variables at coarser spatial scales was also expected, given that fire spread is rapid and is unlikely to respond to topographic variation over fine scales, and that vegetation recovery is likely to be somewhat homogenous within small areas with similar species compositions.

Weather within daily burn perimeters did not play a significant role in predicting NDVI recovery in our models. This result is not unexpected due to the coarse resolution of the data, where the entirety of a daily burn area was attributed with a single value of mean air temperature, humidity, and wind speed. Weather factors at the time of burning certainly play a role in the spread of fire and consumption of vegetation and litter, but in this study weather did not appear to have a strongly significant influence on soil alteration and post-fire recovery. This may be due to the coarse scale of weather data applied to daily burn extents, or because the majority of the study area burned under extreme conditions beyond a threshold where weather may have become more significant.

### Management implications

Warming climates are resulting in a shift toward large, high-impact wildfires occurring at greater frequency throughout western North America [87], and the question of whether bark beetles and wildfires produce compounded effects has important implications for managing to promote ecosystem resilience [88]. Salvage logging has been proposed to mitigate the effects of beetle disturbance and fuel loading on high-severity wildfire. Our results suggest that increased severity of beetle outbreak can negatively impact short-term post-fire vegetation recovery, which may be caused by accumulation of surface fuels. This mechanism may suggest that treatments to reduce surface fuels can promote ecosystem resilience from fire. However, these activities pose a risk toward altering recovery dynamics and facilitating future species composition shifts [89], and may negatively impact long-term carbon storage in forests [90]. Two additional issues suggest that salvage logging would not mitigate the compounded impacts of beetles and wildfire. First, because salvage logging is focused on the removal of dead trees in contrast to fuels on the forest floor, it would not be expected to alter beetle-fire implications for fire characteristics at the soil level. Second, the short period of time in which surface fuels increase the ecological consequences of fire and the highly random nature of wildfires in time and space, implies that salvage logging with the goal of averting the impacts of beetle-wildfire interactions is not a logical management action. Nonetheless, salvage prescriptions have the potential to contribute to other land management objectives in addition to timber production. For example, salvage prescriptions located close to communities in the wildland-urban interface may act as fire breaks and contribute to community and fire fighter safety, and give fire managers confidence in allowing some natural fires to burn.

Impacts of severe beetle outbreak on vegetation recovery also create additional need for enhanced post-fire restoration efforts in areas where outbreak was known to have occurred prior to burning. Our model results indicate that these efforts should prioritize high-elevation, steep, south-facing slopes, as these topographic factors also show a significant effect on vegetation recovery. Restoration of ground vegetation mitigates flooding hazards, prevents soil erosion, and mitigates rising soil temperatures and evapotranspiration potential [91]. Facilitating understory vegetation recovery may therefore prove beneficial for preventing drastic ecological change in severely burned landscapes affected by severe spruce beetle outbreaks.

## Conclusions

Although many studies have tried to determine whether bark beetles lead to larger, more frequent, or more severe wildfires, there have been a number of limitations to determining the true ecological impacts of these overlapping disturbances. Our study quantified pre-fire beetle impacts using the Landsat-derived dNDMI, which likely provides a more accurate measure of beetle severity compared to studies focused on post-fire field measurements. We also used Landsat-based measurements of NDVI recovery to address how beetle-fire interactions may result in compounded effects on surface fuels and soil recovery, and found more conclusive evidence supporting a compounded disturbance interaction compared to studies which have assessed fire severity as a metric of canopy mortality. Future research should focus on long-term examinations of recovery dynamics following wildfires in beetle-killed forests, which will be important for improving understanding of how compounded disturbance interactions from bark beetles and wildfire will affect future forest communities. Additionally, future high-severity fires in beetle-killed spruce forests will need to be studied to determine whether the compounded effects observed in the West Fork Complex are consistent across geographic areas. Although many recent studies have concluded that there is no evidence of a link between beetle outbreaks and increased fire severity, our results indicate that the combined disturbances may result in compounded effects on vegetation recovery.
